# A theory-based evaluation of the Leadership for Universal Health Coverage Programme: insights for multisectoral leadership development in global health

**DOI:** 10.1186/s12961-022-00907-1

**Published:** 2022-09-29

**Authors:** Sophie Witter, Nouria Brikci, David Scherer

**Affiliations:** 1 Institute for Global Health and Development, Queen Margaret University Edinburgh, Edinburgh, UK; 2 Oxford Policy Management, Oxford, UK; 3 Gesellschaft Fur Zusammenarbeit, Bonn, Germany

**Keywords:** Universal health coverage, Leadership development, Adaptive challenges, Africa, Asia, Theory-based evaluation

## Abstract

**Background:**

Leadership to manage the complex political and technical challenges of moving towards universal health coverage (UHC) is widely recognized as critical, but there are few studies which evaluate how to expand capacities in this area. This article aims to fill some of this gap by presenting the methods and findings of an evaluation of the Leadership for UHC (L4UHC) programme in 2019–2020.

**Methods:**

Given the complexity of the intervention and environment, we adopted a theory-driven evaluation approach that allowed us to understand the role of the programme, amongst other factors. Data from a range of sources and tools were compared with a programme theory of change, with analysis structured using an evaluation matrix organized according to the Organisation for Economic Co-operation and Development–Development Assistance Committee (OECD-DAC) criteria. Data sources included key informant (KI) interviews (89 in total); surveys of the 80 workshop participants; a range of secondary data sources; case studies in two countries; and observation of activities and modules by the evaluator.

**Results:**

Participants and KIs at the global and country levels reported high relevance of the programme and a lack of alternatives aiming at similar goals. In relation to effectiveness, at the individual level, there was an increase in some competencies, particularly for those with less experience at the baseline. Less change was observed in commitment to UHC as that started at a relatively high level. Understanding of UHC complexity grew, particularly for those coming from a non-health background. Connections across institutional divides for team members in-country increased, although variably across the countries, but the programme has not as yet had a major impact on national coalitions for UHC. Impacts on health policy and practice outcomes were evident in two out of seven countries. We examined factors favouring success and explanatory factors. We identified positive but no negative unintended effects.

**Conclusions:**

While noting methodological constraints, the theory-based evaluation approach is found suitable for assessing and learning lessons from complex global programmes. We conclude that L4UHC is an important addition to the global and national health ecosystem, addressing a relevant need with some strong results, and also highlight challenges which can inform other programmes with similar objectives.

**Supplementary Information:**

The online version contains supplementary material available at 10.1186/s12961-022-00907-1.

## Background

Leadership and management are recognized as important enablers for improving programme performance, strengthening health system capacity, enhancing connections with target populations, increasing the ability of health systems to respond effectively to change and, at a high level, achieving country ownership of health policy goals [1]. These imply considerable skills, for which there is currently limited training and development support [2].

The core premise underpinning the Leadership for Universal Health Coverage (L4UHC) programme, which has been developed from 2014 onwards, is that universal health coverage (UHC) [3] has both technical and adaptive challenges. Technical challenges can be addressed through information, factual assessment and subject knowledge, but adaptive challenges can be more challenging, as they typically involve confronting the status quo to change behaviour, practices and ways of working which have been established.^1^ Capacity development work for the specific technical challenges has received attention; prominent examples of initiatives by international development partners are the World Bank Flagship Course on Health Sector Reform and Sustainable Financing [4] and the WHO Advanced course on health financing [5]. Less has been done to develop leaders’ capacities to address the adaptive challenges, although it is widely recognized that UHC has political leadership at its core [6]. The L4UHC programme aims to address this gap by enabling senior decision-makers to develop the leadership skills and collective actions needed to take forward UHC in their countries [7].

A number of leadership development programmes exist or have recently been implemented. These include high-level, cross-country initiatives such as the Harvard Ministerial Leadership in Health [8] or the Aspen Institute Ministerial Leadership Initiative for Global Health [9], which aim at the highest ministerial ranks and focus on technical cooperation between health and finance ministries or on predefined aspects of reforms (such as efficiency improvement, reproductive health or general health financing strategies). Others provide cross-country leadership training focused on health teams from each country, such as the Management Sciences for Health Leadership, Management and Governance Project [10] or the Yale Global Health Leadership Initiative [11]. There are also networks to support health leadership collaboration across countries [12] and national-level leadership programmes, such as the health leadership programme in South Africa [13], which tend to focus more on clinical leadership, and district- or hospital-based training and mentoring schemes [14]. Other leadership development programmes are multisectoral, such as the Tony Blair Africa Governance Initiative [15] or the World Bank’s Global Partnership on Collaborative Leadership for Development [16]. However, the L4UHC programme is distinct in that it provides a programme to enhance UHC leadership that:

- creates learning opportunities across countries within a region;
- focuses on UHC but involves key stakeholders, not just from health but also political bodies at the federal, regional and local levels within supported countries, as well as the private sector, civil society and development practitioners, aiming at building national coalitions; and
- focuses on adaptive leadership skills.

In this article we report on an evaluation of the L4UHC programme, which was conducted in 2019–2020. The evaluation literature for leadership development programmes in global health is limited, given methodological challenges, the limited number of such programmes and the diversity of their aims and approaches, as well as lack of resourcing for evaluations, especially in low- and middle-income settings [17]. It is therefore important to share methodological reflections and also substantive insights, to increase transparency and foster learning in this field.

This paper reports formative and summative evaluation results, examining whether inputs, outputs, outcomes and impact occurred as expected, but also probing the more exploratory learning questions around what worked, what did not work (and why), the conditions under which L4UHC is likely to be effective, and how it, and programmes with similar objectives, can be tailored to maximize their chances of success.

Box 1. Overview of the L4UHC programme L4UHC is implemented under the stewardship of a L4UHC Global Steering Committee, composed of the Gesellschaft für Internationale Zusammenarbeit (GIZ, German Society for International Cooperation), Swiss Agency for Development and Cooperation (SDC), the World Bank, WHO, United States Agency for International Development (USAID) and Expertise France, linked through the Partners for Health (P4H) Network. In Asia, L4UHC is implemented in cooperation with the Asia Pacific Network for Health Systems Strengthening. There are multiple stakeholders within these partner organizations, including at the head office and country levels in participating countries.

Each L4UHC cycle is led by the L4UHC management team, supported by country focal persons (CFPs), in-country coaches and/or regional facilitators.

To facilitate the implementation of the programme, a L4UHC CFP is designated in each participating and host country. The CFPs liaise with the P4H partners and the national actors and provide country briefings that provide insights on the state of UHC reforms. In the participating countries, CFPs support the selection and briefing of the country teams. In the host countries, they support the learning exchange with the host country.

The learning methodology was originally informed by the Akademie für Internationale Zusammenarbeit (Academy for International Cooperation)’s Leadership for Global Responsibility approach [18], which emphasizes the importance of the “inner condition” of leaders and core capacities of innovation, transformation and cooperation. It also drew on elements from the Rapid Results Approach [19], which has been used successfully by the World Bank.

The cycle starts with a 6-month preparation phase, including working with CFPs, preparation of activities and identification of participants. Participants are expected to be high-level UHC stakeholders from government, the private sector and civil society (e.g. ministers, director generals, members of parliament and chief operating officers). There are generally around 10 participants per country team, and three to four country teams per cycle.

The core learning activities take place over 1 year, in principle, and include:

1. peer-to-peer exchange in host countries (with participant countries attending modules in three different countries in the region): the first module focuses on individual leadership capacities; the second on collective action (change management and planning interventions for rapid results in each country); the third on committing to key actions; during activities, resource people bring leadership mentoring and technical expertise in UHC;
2. activities including immersion in host country experiences;
3. participants working as a country team between modules, supported by in-country coaches: in the first practical action phase, they meet as a team to do stakeholder mapping and meet with other UHC stakeholders to understand wider perspectives; in the second phase, the focus is on implementing agreed short-term initiatives; and short tailored workshops held on specific UHC issues as required throughout the year.

During the follow-up phase, P4H partners remain in touch to support the team’s momentum, checking in after 6 months but with indefinite potential follow-ups in theory.

## Methods

### Evaluation approach

Measuring the outcome of capacity development programmes with respect to change in leadership competencies is difficult [20]. Measuring the capacities of individuals and organizations tends to be subjective, and the intended transformation in the participants of a programme has different aspects, ranging from new analytical tools through to interpersonal skills and personal reorientation. Not all of these changes are testable or would manifest themselves in the short term. Participants’ leadership skills are influenced by myriad factors, and there is unlikely to be a linear correlation between one activity and the overall personal transformation. At the same time, a multitude of other factors can influence programme participants over the duration of a programme, especially when it lasts 1 year or more, as this programme did. This makes it difficult to attribute any changes observed in individual participants directly to the capacity development programme. In addition, and unlike many other capacity development programmes, L4UHC does not work with pre-existing teams, whose later functioning can be observed. Instead, it works with individuals drawn from a range of different organizations in their home countries (see Box 1).

The same issues are an even greater concern if we go beyond individuals to examine impact on entire reform processes. Trying to isolate the contribution of a leadership development intervention in dynamic contexts is extremely hard. Leadership is only one component of the actions necessary to achieve UHC—some of the others being technical expertise, overall successful multisectoral action and the creation of fiscal space [3].

Our overall approach was therefore one of contribution analysis [21]. We aimed to understand the role of L4UHC in a dynamic context and as one of many changing elements, which implies a good grasp of the country contexts and how those change over the period of the programme.

A study that scanned evaluations of 55 leadership development programmes [17] highlights the importance of developing a theory of change, of integrating evaluation into the learning programme, and of going beyond individual and team assessment of impact to understand behaviour change and systemic impact. We attempted to reflect those priorities in our approach.

Our approach used mixed methods, which is recognized good practice given the complexity of indicators and measurement [22] and the need to combine sources and triangulate evidence where possible. Internally generated data from the programme were used where possible.

As the period of evaluation was too short to allow us to collect robust evidence on changes at impact level (UHC indicators such as population coverage, equity, financial protection and quality of care), our focus was on analysing inputs and changes in outputs, intermediate and higher-level outcomes, and their explanatory factors, using the structure of the Organisation for Economic Co-operation and Development–Development Assistance Committee (OECD-DAC) criteria (relevance, effectiveness, efficiency, impact and sustainability) [23].

### Theory of change

**Fig. 1:**
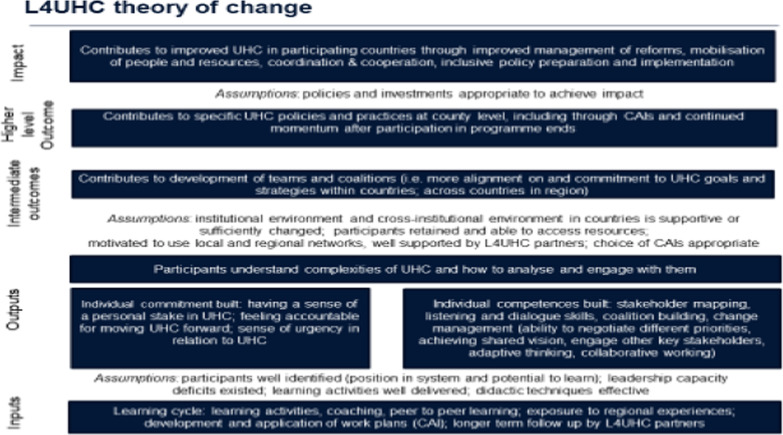
Evaluation theory of change for L4UHC

Working from core programme documents, such as the implementation manual [24], and in collaboration with programme staff, we developed a theory of change against which to assess programme contribution (Fig. 1). This was developed in December 2018, at the start of the recent programme cycle, in combination with programme partners.

The theory of change outlines the L4UHC inputs, which include experiential and intellectual learning and iterative practice. Under certain assumptions—including that the need for capacity development for L4UHC was real, that participants are well-identified, that learning activities are well-delivered and that their techniques are effective—these will contribute to key outputs at individual level.

Direct outputs of the activities include increased commitment, improved competencies and understanding of UHC. Commitment includes having a sense of a personal stake in UHC; feeling accountable for moving UHC forward; and having a sense of urgency in relation to UHC. Competencies include stakeholder mapping, listening and dialogue skills, coalition-building and change management (the ability to negotiate different priorities, achieve a shared vision and engage other key stakeholders, as well as to engage in adaptive and innovative thinking and work collaboratively). These feed into the learning objectives for participants of understanding the complexities of UHC and how to analyse and engage with them.

An important intermediate outcome is the development of coalitions, which can operate within countries but also across the region, while the higher outcome focuses on implementation of the agreed country action initiatives and follow-through on reforms. Important factors to investigate that will enable or block these include the institutional and cross-institutional environment, participants’ ability to access and negotiate resources, and their motivation to use local and regional networks established through the programme. While L4UHC focuses mainly on individual leadership development, we also examined potential spill-overs into changed organizational culture. This was not directly incorporated into the theory of change but was desirable, as it would ensure sustainability, even when staff move posts.

Ultimately, the expected impact was increased progress on UHC, which should follow if policies and programmes were appropriate to achieve impact.

Based on the programme design, its theory of change and the evaluation approach laid out, we developed an evaluation matrix highlighting the key evaluation questions and how these would be answered (linking to the different data sources, their frequency and analysis approach: Additional file 1).

## Research tools

### Key informant interviews

Key informants (KIs) were purposively selected to bring insights from a number of constituencies, including:

- programme participants
- CFPs supporting them within this programme
- L4UHC core management team and partners
- course facilitators
- country host organizations
- country-based coaches
- some external KIs who were knowledgeable about UHC but not directly engaged in the programme (in the two case study countries).

Participants were identified at the time of the first modules, aiming for a range of profiles within each team and to reach a substantial proportion of each. The aim was to follow up with final interviews of the same cohort in the last module.

All respondents were sent an email by the lead researcher, which gave information on the evaluation and asked for their oral informed consent. Interviews were conducted in private settings—in person for the first module, but entirely virtually (by WhatsApp, Skype or Teams) for the final module, as the last modules were not held face-to-face due to COVID-19 restrictions.

Interviews followed a semi-structured question guide that followed the evaluation matrix structure. Conversations took place in English in the Asia cycle and in French for the Africa cycle. They were recorded and noted for later thematic analysis, based on the evaluation matrix but allowing for inductive coding as the material prompted. Interviews lasted from 30 minutes to 1 hour, with most taking 45 minutes.

The summary of the KIs interviewed is provided in Table 1. The bulk of the interviews come from participants (50), of whom more were drawn from Africa, but this partly reflects the larger number of teams participating in the programme from that region. Overall, we reached 45% of participants (slightly below the 50% target at the start). The larger number of men interviewed in this group also reflects patterns within the teams, which are discussed in the findings below. Other constituencies were well-represented, although the group of external informants (those not involved in the programme and who were reached through the case study interviews) was limited. We discuss this in the limitations section.

**Table 1:** Summary of evaluation KIs

	Male	Female	Total participants	% of total for that team	Total interviews
Asia	8	6	14	40%	21
Myanmar	3	3	6	46%	8
Pakistan	2	2	4	31%	7
Viet Nam	3	1	4	33%	6
Africa	14	8	22	49%	29
Burkina Faso	2	3	5	50%	8
Cameroon	4	1	5	38%	6
Niger	4	–	4	36%	5
Senegal	4	4	8	73%	10
Total participants	22	14	36	45%	50
External informants (country case studies)	3	3	6	–	6
Myanmar	1	3	4	–	4
Senegal	2	–	2	–	2
Core management team and P4H partners	5	3	8	–	11
Country and regional focal people	6	2	8	–	14
Module facilitators	2	1	3	–	3
Coaches	1	2	3	–	3
Country hosts	1	–	1	–	1
Organizers	–	1	1	–	1
Total non-participants	18	12	30	–	39
Total	40	26	66	–	89

Differences in KI numbers and interview numbers reflect the fact that many of the cohort were interviewed at the start and at the end of the programme

In order to protect the identity of interviewees in relation to citations, we have used very broad labelling, indicating whether the KI was global or from a specific country. Specific countries indicate participants; coaches and CFPs are included in the global KI group (although some are attached to countries, indicating this would reveal identities).

### Participant surveys

Surveys were administered at the end of each of the three modules and in each of the two regions (six rounds in total). The questions focused on the participants’ perceptions of the preparation for the module, its organization, the components within it, and overall perceptions and recommendations. For Modules 2 and 3, there were additional questions on the practical phase between modules, including an additional review meeting organized in July 2020. The survey was tailored for each module and also translated into French for the Africa cycle. All participants were given an hour on the last day of each module to fill in the survey, which largely contained closed questions, using a Likert scale, as well as open-text questions for more exploratory topics (such as suggested improvements to the modules).

The analysis was largely quantitative, with thematic inductive coding of open responses by the lead evaluator.

Table 2 presents the survey respondents by country and module. Here it can be seen that the response rate was good in general—an average of nine respondents per module and team (where team size ranged from 10 to 13 across the countries), with an overall proportion of just under 80% (but dropping between Module 1 and Module 3).

**Table 2:** Survey respondents, by country and module

	Module 1	Module 2	Module 3	Total
Burkina Faso	9	5	7	21
Cameroon	12	12	7	31
Niger	9	10	7	26
Senegal	7	9	8	24
Myanmar	12	10	9	31
Pakistan	9	9	9	27
Viet Nam	13	9	7	29
Total	71	64	54	189
Respondents as % of total group	89	80	68	

Table 3 presents responses by sector and profile of participants. The largest number of respondents were from the Ministry of Health (MoH) and other ministries or public sector organizations, followed by civil society and the private sector. In terms of profile, senior management dominated. This reflects the participant profiles.

**Table 3:** Survey respondents by sector and profile (per module and region)

	Module 1		Module 2		Module 3		Total
	Africa	Asia	Africa	Asia	Africa	Asia	
*By sector*							
Civil society	6	4	1	4	4	2	21
MoH	12	18	10	15	6	13	74
Other ministries or public sector	18	8	22	7	11	5	71
Private sector	1	4	1	2	2	3	13
None of the above			2	0	6	2	10
*By profile*							
Mid-management	0	11	0	10	2	9	32
Operational staff	4	8	3	9	2	4	30
Senior management	33	15	32	9	20	11	120
None of the above	0	0	1	0	5	1	7
Total	37	34	36	28	29	25	189

### Secondary sources

Secondary sources were identified throughout the programme according to whether they shed light on the questions in the evaluation matrix, and they were analysed using that structure. They included:

- data generated by the participants in the course of the programme, such as plans for collective action initiatives (CAIs), reports on activities and plans, group presentations, feedback at end of day during modules, and L4UHC programme reports;
- reports by coaches and CFPs in meetings (orally and as shared through presentations and notes);
- budget and expenditure data from the programme coordinator; and
- country documentation, such as national health plans, reviews, evaluation, political economy analyses, health system assessments, donor mapping and routine health information system data (for the two case study countries—described below).

In addition to these secondary sources, the lead evaluator took notes of observations during Module 1 and also during online meetings thereafter.

### Country case studies

Two countries were chosen for more in-depth analysis—one in the Asia region (Myanmar) and one in Africa (Senegal). Both were chosen purposively in consultation with the L4UHC team. The selection criteria included having a dynamic UHC policy environment; having sufficient L4UHC country team participant numbers; and engagement of P4H partners in-country. In these two countries, more structured extraction of data from background documents and in-country interviews with stakeholders outside the L4UHC participants aimed to allow for more contextual depth on UHC challenges, progress, and the contribution of L4UHC and other factors.

The country case studies were originally scheduled to follow Module 3. However, due to delays to the last module caused by COVID-19 and the related travel restrictions, they were undertaken remotely in August and September 2020. Apart from analysing national UHC documents, six additional interviews were conducted for Myanmar, of which five were external to the programme but engaged with UHC at the country level. For Senegal, six additional interviews were undertaken, of which three were engaged in L4UHC and three were external. An adapted topic guide was used for these interviews, which were undertaken in a similar manner to the KI interviews above.

### Data analysis and reporting

Framework analysis, using deductive and inductive approaches and guided by the evaluation questions (Additional file 1), was used by the lead researcher to analyse qualitative data, such as KI interviews (which were recorded and then summarized), open-ended survey responses, and programme and policy documents. Survey data were entered into SurveyMonkey (either directly by participants or by the research team based on paper copies of the surveys) and analysed using Stata and Excel, disaggregating responses by country, region, gender and sector of respondents. All findings were integrated using the evaluation matrix structure, based on the OECD-DAC criteria and main evaluation questions (which followed the theory of change). Findings were shared with the L4UHC management team and wider partners for comments and corrections in December 2020.

### Ethics

Oxford Policy Management’s ethical review committee reviewed the evaluation protocol and tools, and approval was given early in 2019 before data gathering began. As all participants were high-level representatives with good comprehension of concepts, we did not anticipate any major risks. All participants were provided with information about the study and were assured of privacy and confidentiality of reporting. All were asked for consent by email and reminded that they could leave the evaluation process at any time, without needing to give justification.

## Results

### Overview of programme and context

The 2019/2020 cycle included two in-person modules, two practical phases, and a final, semi-virtual third module, which was adapted in format and held later than planned due to COVID-19. The programme covered Myanmar, Pakistan and Viet Nam in Asia, and Burkina Faso, Cameroon, Niger and Senegal in Africa. A total of 80 participants were divided into country teams of 10–13 people. Over 60% were men, which reflects leadership patterns in the countries concerned (e.g. for Pakistan, Niger and Cameroon). In relation to constituencies, participants came from a variety of backgrounds, as desired, though the patterns varied by country team. The MoH and relevant social agencies (e.g. ministries of social protection or community development) were well represented. However, the Africa region mobilized cross-cutting government representatives, such as the ministry of finance, the prime minister’s office, the presidency, and also parliamentarians to a greater extent, with interesting disciplinary additions such as lawyers and journalists. The private sectors (for-profit and not-for-profit) were relatively poorly represented across the board.

Contextual factors influencing programme implementation and results included, most importantly, the COVID-19 pandemic, which interrupted CAIs in some settings, distracted senior staff and caused the final module to be delayed and then to be held semi-virtually (with some country teams meeting in person but regional connections made online). COVID-19 may however also open some opportunities by raising the profile of the health sector nationally, and also normalizing virtual interactions, which potentially opens new modalities of interaction for the future. Other important contextual factors noted included the ongoing insecurity in West Africa, which affected priorities in some of the participant countries (such as Burkina Faso), national elections in three participant countries, which may increase turnover of participant posts, and growth in the P4H network, which may increase sustainability of the programme.

### Relevance

We examined the extent to which L4UHC addresses priority needs at individual participant and country levels—in other words, whether it addresses a priority gap or bottleneck that is not already being met from other sources. Data to answer this question were drawn from the first set of participant interviews, as well as interviews with CFPs, core team members and external KIs in case study countries.

Participants generally saw leadership as important to them individually, and few had access to comparable training. Adaptive leadership was also highlighted as an important bottleneck for UHC at the country level by global KIs, participants and secondary sources.

*I think there is an appetite for this, if delivered in a way that is relevant to people’s work. The work that these people are doing often comes with minimal feedback and support. It is important to our long-term development.* (Burkina Faso KI)

*Leadership is really an issue at all levels. You don't just need a presidential decree to make things happen. … It requires multiple actors, including civil society, but only the state can guarantee sustainability (rights and regulations). We need to find a way to dialogue and reach consensus. We have talked about UHC for 10 years but are not getting far. This needs everyone to work together.* (Global KI)

In relation to the question of whether L4UHC duplicates other programmes, a few candidate programmes were identified but all have distinctive approaches. There was thus a consensus that L4UHC offers something that potentially adds value and is complementary to other efforts.

### Effectiveness

We examined the extent to which L4UHC has met its objectives at output and outcome level, as well as how these were achieved and for whom, and what factors were important (positively or negatively), both from the programme side and externally. This section draws on all main data sources—participants and wider KIs, the survey, case studies and secondary sources.

### Outputs

Our first section focuses on the individual level, examining the evidence for changes in commitment, competence and understanding by programme participants, followed by a discussion of explanatory factors relating to the way the programme was run and feedback on the various components. This section draws on all evaluation data sources, but especially on survey data and interviews.

Individual commitment to UHC was already reported as high for most participants at the start of the programme—in that sense, selection was good, and some had long careers supporting UHC—which meant there was less scope for gain respecting this dimension. Change in commitment for some individuals was noted, however along with growth in understanding.

Individual competencies were considered in relation to leadership, such as ability and confidence in areas including stakeholder mapping, listening and dialogue, coalition-building and change management. Open answers from the three module surveys highlighted listening skills as one of the main competencies participants felt they had gained across both regions, with coalition-building increasing by Module 3 (Table 4).

**Table 4:** Top skill gained (Module 3 participant presentations)

Skills	Burkina Faso	Cameroon	Niger	Senegal	Myanmar	Pakistan	Total
Deep listening	2	3	6	3	6	6	26
Managing adaptive challenges	1	3	2	–	3	3	12
Creating coalitions	–	2	2	3	–	5	12
Stakeholder mapping	4	–	–	1	2	2	9
Self-management	1	1	–	–	3	1	6
Total respondents	8	9	10	7	7	10	51

Analysis of participant interviews at the start and end of the programme indicated that, in many cases, participants had considerable skills in leadership relating to their ongoing roles at the start, but all had been able to develop or deepen these skills in some way. Unsurprisingly, some of those who were less experienced at the start reported learning the most. Feedback from those working with the groups also suggested improvements in listening and dialogue, including increase respect for minority positions and greater involvement of those with lesser perceived status at the start of the programme.

Regarding the understanding of UHC, slightly different patterns emerged from Asia and Africa, which may be explained by the composition of the groups. There was more evidence of a development of understanding in the Africa group as a whole (though there are individual examples from Asia). This may be because the participants in Africa came from more diverse organizational backgrounds, which meant some had a more limited starting understanding of UHC.

Most participants in Asia started with a good basic understanding of UHC, although it was closely equated with health insurance in some settings, which can limit a broader understanding of the concept, the different routes that it can take and its complexity. For some individuals with lower starting levels, we identified growth in comprehension, but this was not clear across the board.

### Explanatory factors

We considere how the output changes noted above were achieved, what worked well (or not) and what lessons emerged. We examine evidence relating to the assumptions in our theory of change at this level—for example, how well components were delivered and how well participants were selected—as well as the role of external factors, such as country contexts. Although these are discussed under outputs, given the connections across the theory of change, they also influence higher levels in the theory of change, such as outcomes and impact. Some key findings are highlighted here.

#### Overall design

This was seen by participants and facilitators as good, but with some tensions perceived between a focus on individual personal development and a focus on teams achieving concrete results in their action plans, and also (especially initially) on the balance of adaptive and technical content. Some participants were also not clear on the approach of L4UHC at the start of the programme, but appreciation grew with understanding.

*I really like the three modules and the design, starting with not heavy topics, [which] makes participants increase their interest and motivation, and later try to integrate with more technical aspects.* (Myanmar participant)

#### Country selection

In general, informants felt the countries in Africa had been appropriately selected—all faced different challenges, but there was room in each for benefits from participation. Less feedback was received on the Asia country selection.

## Participant selection

L4UHC aims to target senior individuals in a range of UHC-related institutions, who have time to attend and participate consistently. Overall feedback on the make-up of teams was positive, although some gaps were noted in each case by participants.

*They chose participants well—hard-working, activists, well-motivated to improve life in Niger, also representing all sectors.* (Niger KI)

Gaps related to a variety of factors, including (from the programme side) the institutional links that L4UHC partners in-country have and how they mobilize them (or not), as well as overall limits on numbers of participants and financing constraints. From the country side, the need for language skills, time availability of senior staff, and internal hierarchies and procedures were influential.

## Continuity of participation

Continuity of participation in modules was good in general. For CAIs, it was more partial, with many teams led by an active core of a few participants.

## Host countries

Views on these were largely positive, with some reservations expressed on the sites in Asia.

## Preparation pre-programme

Thoroughness in preparing candidates for the start of the programme varied across the countries (from very good to very limited).

## Modules overall

Overall, participant satisfaction with the modules was high—close to 60% rated the modules as excellent or good overall, with satisfaction highest for Module 2 and lowest for Module 3 (which can be understood in relation to the disappointment regarding this module moving from in-person to semi-virtual due to COVID-19). Considerable variation was shown across countries, from 25% in Viet Nam to 93% in Pakistan (Table 5).

**Table 5:** Overall rating of modules (% rating them excellent or good), by country

	Burkina Faso	Cameroon	Niger	Senegal	Myanmar	Pakistan	Viet Nam	Average
M1	88.9	41.7	88.9	57.1	63.6	88.9	23.1	64.6
M2	60	77.8	100	33.3	70	100	55.6	71.0
M3	14.3	50	83.3	42.9	22.2	88.9	No data	43.1
Average	54.4	56.5	90.7	44.4	51.9	92.6	26.2	59.5

Scores represent the proportion rating the module overall as excellent or good in the end-of-module surveys. The Viet Nam team did not attend Module 3, hence the data gap. Averages have been adjusted for this

It is also interesting to examine these scores by the profile and sector of participants. Senior management had the highest overall rating (56% rating the modules as excellent or good), compared with 51% for operational staff. Across the sectors, the private sector gave the highest ratings of excellent or good (71%) and civil society had the lowest (46%), although we need to note low numbers in both these categories. Another overall satisfaction metric was the proportion of participants who would recommend the programme to others. The average here was high, at just under 85%.

## Module content

Participants were asked about each component in the modules, on four domains—whether the exercise was engaging, whether its delivery was well-paced, whether the content was relevant to them, and whether they had learned something substantive they felt they could apply. Aggregate scores indicated high overall satisfaction, but more so in Africa than Asia. Surveys and interviews suggest that participants most enjoyed the participative and experiential components, as well as those which allowed for sharing across settings and teams.

After some debate about adapting the format to COVID-19 restrictions, the regions met virtually for Module 3, though with some country teams meeting in person in one place, where this was possible. This allowed for some intimacy, although of course disappointment was expressed by participants because the full modules were not held. Survey responses, aggregating across all components in the module, were positive across both regions, though there was a perception by a significant minority of lack of interactivity. One country delegation did not choose to join this final module.

## Organization/facilitation

Overall, modules were seen as well-facilitated and structured, although responses varied by module and country, and there were some dissenting voices for Module 3 in particular. A large proportion were satisfied with the practical arrangements, but there were a variety of patterns across countries and modules, with more dissenting voices in Africa (relating to per diems and travel).

## Practical phases

Between the modules, participants were encouraged to undertake activities as a group (including stakeholder mapping, sensing journeys and other consultations) to develop preliminary ideas for their CAIs in phase 1 and then to implement them in phase 2. When questioned about these activities in the survey, most people reported undertaking them and finding them useful; a lower proportion felt they had had sufficient support from their CFPs, particularly in two countries.

## CFPs and coaches

In general, the role of CFPs appeared to be more central in the Africa cycle. Clear roles were played in relation to each country and there was consistency and reasonable intensity in the support provided by the nominated CFPs (who combined this role with being the local P4H focal points). Perhaps partly as a result, the role of coaches was weaker in this region, which was not the case in the Asia cycle. In Myanmar, the roles of the P4H focal point, the L4UHC CFP and the local coach were assigned to one person. In some settings, such as Pakistan, there was a perceived overlap between the role of the CFP and the role of the coach. In Asia, the regional coach helped provide momentum and support country-based coaches.

## External factors

The COVID-19 pandemic was a major factor, reducing personal contact in the second practical phase and in Module 3, while also absorbing participants with more urgent tasks.

*The major challenge is the attention span, which has reduced dramatically with COVID-19. Also, online digital meetings are the opposite of building a real trust and deep collaborative spirit within a team. This presents a real challenge for L4UHC and [for] building personal relationships.* (global KI)

Country buy-in was also important but was notably absent in Viet Nam. Cultural assumptions about leadership, P4H partners’ alignment in participant countries, and language barriers were all noted as important external factors influencing the programme results.

## Intermediate outcomes

For these, we drew mainly on KI interviews with participants and other stakeholders.

### Team development

Global KIs perceived a substantial team-building effect for participants in several countries, although this was varied across teams and regions.

*They often arrive at the table with a lot of distrust across organizations. They then gradually move from talking from their organizational perspective to talking about the team.* (global KI)

This is a domain where we observed considerable change, especially in relation to the teams in Africa, where strong bonds within the group appear to have been formed in some of the teams, despite (or maybe because of) their diverse institutional backgrounds.

*The programme helped create a team; it didn’t however completely manage the conflicts but [it] did make them more explicit. The whole team could see them and discuss them. So not a perfect dialogue, but the dynamic helped to manage them within the “family”. The dynamic was good—everyone was active and applied. … A real sense of being a family!* (global KI)

The teams in Asia presented a more complex picture in that the Viet Nam team did not appear to have worked together in a coordinated way except through other national fora. In Pakistan, strong teamwork emerged, but centred around two CAIs more than at whole-team level. In Myanmar, collaboration was emerging but perhaps at early stages, also given the interruptions of COVID-19. The course structure and the diversity of teams were seen as enablers of change in this domain, while the constraints of hierarchy were barriers to equitable group work, especially in some settings, like Pakistan. Some increase in team members’ wider networks was also reported across all countries.

### National coalitions

More ambitious than team-building, or building links between team members, was the question of whether L4UHC has contributed to a wider shift in national coalitions working for UHC. This could have been through engaging other actors in their meetings, their CAIs or other activities. In general, this was less reported on by stakeholders, so the findings here are limited, and it may be that this is too ambitious an expectation for this stage in the programme. Limited change in national coalitions was found in general, but in some settings, such as Burkina Faso, the group may be helping to reduce tensions between institutions over mandates, as well as increasing the capacity of individuals within existing national networks.

*There was already a multisectoral group working on UHC before, so that hasn’t changed much, but L4UHC has reinforced the capacities of those in the group who were in that multisectoral forum.* (Cameroon KI)

### Regional networks

A second aspiration within the theory of change was for the L4UHC programme to strengthen regional UHC networks, which could occur via strengthening of the links of individuals or teams to wider networks, or by creating a network across L4UHC country teams. There was weak evidence for change in this respect in this cycle. National grouping appeared to be prioritized in group work in modules, and there was no encouragement specifically to communicate across countries between modules. Some personal relationships were established across teams, but these were limited. KIs agreed this could be given more explicit priority in future.

### Higher-level outcomes

We examined the impact of L4UHC on policy and practice in participating countries, particularly though the lens of the CAIs. Overall, there was good engagement in significant national processes by the Africa teams, but limited concrete results where a clear contribution of the L4UHC team could be traced as yet, with the exception of Burkina Faso, where the group appeared to have facilitated important progress on universal health insurance development and the response to COVID-19. The Burkina Faso team chose to focus their CAI on improving the operation of the policy to cover indigents in the UHC programme, including supporting the creation of a shared platform to coordinate actors and ensure full coverage of the country. In June 2020, the group reported that contracts had been signed with implementing organizations and that the L4UHC team had supported awareness-raising around the new policy. Results reported in October 2020 included the launch of the policy to cover indigents (in September 2020).

In Pakistan, the team was able to achieve concrete gains in two areas—increasing enrolment in the national health insurance programme in Rawalpindi district by novel and networked approaches to enrolment (growing from 40 to 59% coverage over summer of 2020); and setting up a draft memorandum of understanding between the State Life Insurance Corporation of Pakistan (the main insurer working on UHC) and Indus Hospital and Health Network, a major private not-for-profit hospital network, to increase the network of UHC providers—while the CAIs were still ongoing with few results to show as yet in Myanmar, and no progress was seen in Viet Nam.

Factors favouring success included having clearly defined CAIs; having CAIs with achievements linked to capacities held by group members; working with the grain of political priorities; and having a strong team dynamic drawing from diverse group skills and networks. Constraining factors included the impact of COVID-19, lack of resources to support team activities and unsupportive institutional contexts.

### Efficiency

We examined the overall costs of L4UHC, make judgements (insofar as it is possible to do so) on its value for money and identify some potential areas for efficiency gains. Evidence for this came from the L4UHC financial reporting and also from KI interviews with the management team.

The two core funders of L4UHC were SDC and GIZ. They cofinanced the core costs of L4UHC, including the cost of the full-time global coordinator, the events costs (running of the modules), the costs of the coaches, and the monitoring, evaluation and communications. Smaller contributions came from WHO, for the time of core team members; from the World Bank, for the time of the didactic lead and also for some country delegations (Niger in this round); and from USAID and Expertise France, mainly covering the time of their staff, who provide time inputs to support the programme.

The overall cost of the programme for this cycle was just over €2.1 million for the full programme, which equated to €26,705 per participant. This cost included all the staff and travel time provided by partner organizations to support the programme (commonly omitted from costings). The largest element was the event costs—the running of the modules—which absorbed around 43% of overall resources.

The programme was relatively expensive compared with short training programmes, but the L4UHC programme takes a longer and more ambitious approach, and there are few good comparators/benchmarks by which to judge its value for money. Making a judgement on value for money is challenging for a programme like L4UHC, which has no direct comparators and with outputs and outcomes that are multiple, potentially long-term, and in some part intangible. Potential gains over time through reforms to improve health, health access, social equity, and save waste in the health sector of participating countries could easily repay investments many times over. Equally, these gains are not guaranteed, and longer-term outcomes are unpredictable.

As important as judging value for money may be identifying ways of increasing its efficiency, for example by establishing it as an independent entity to reduce overhead costs, streamlining decision-making, and clarifying roles and responsibilities within it.

## Impact

We examined the likely longer-term impact of L4UHC and any unintended effects that had been observed (positive or negative). The evidence here drew on KIs across different constituencies.

In Asia, there was limited optimism about impact in Viet Nam. In Myanmar, there was judged to be a possibility of improved longer-term collaboration across actors on UHC, although quite strong central control over key decisions make this challenging in the short run (and the military coup in 2021 has not helped). In Pakistan, there was strong momentum for extension of health insurance, which is likely to continue, with L4UHC having contributed to strengthened personal networks for some actors to support this movement.

In Africa, some KIs saw a shift in attention to those in most need, to which L4UHC may have contributed. It is plausible that the cross-sectoral teams established in all four settings may play catalytic roles in future policy development, funding and implementation. Consideration of how to maintain the teamwork established to date in L4UHC is now required.

Risks to impact come from changing posts and staff turnover for participants, but also from shifting political agendas and lack of policy continuity.

KIs were asked whether they had observed any negative unintended effects, but none was raised. On the positive side, however, a number of unintended effects were noted:

- A resource for responding to COVID-19—for example, in Burkina, one KI pointed out that, as the group was already in existence, it was able to support better communication and more harmonized messaging with the population across different actors.
- In relation to their role as P4H focal points, two CFPs stated that their work on the programme had extended their networks and contacts, with side benefits for their work for P4H.
- Personal development for resource people—although the programme understandably focuses on participants, some of those helping to organize and run it also reported some personal benefits in terms of changed perspective and heightened skills (e.g. in facilitation).
- Career development—helping participants progress their career was not a direct aim, but it was pleasing to hear at least one concrete example of a participant who had been promoted and who attributed this to growth in skills and confidence supported by the programme.
- Side benefits to older participating cohorts—members of the Nepal and Chad teams who were alumni of the programme joined some activities in 2020, which aimed to provide inspiration for the current cohort but also to stimulate continued actions by those teams. We did not examine the extent to which this was effective, but note this as a potential positive effect.

In addition, we found some evidence of benefits from participation for the participants’ organizations. This was not a central expectation in the theory of change because of the individual and cross-organizational design of L4UHC. The main mechanisms for transmitting benefits were as follows: the person being more effective in their core work role (skills, confidence, networks); sharing learning (substantive, also on L4UHC methods) from the programme with colleagues; and strengthening their engagement in other activities (e.g. voluntary and domestic).

*After the course, my boss acknowledged my role and involved me more in decisions. This did not used to be the case, but I am now involved in policy-making.* (Pakistan KI)

## Sustainability

Finally, we considered how likely it is that the benefits noted from L4UHC would continue after the end of the programme—which reflects commitment by various parties, capacity and also affordability—and how sustainability could be enhanced. This section draws on KI interviews, and also on participant presentations in Module 3.

Overall sustainability varied by level. In relation to individual commitment, this is likely to remain high, with benefits for the country, depending on future postings. The team dynamic appears to be more secure in Burkina Faso than in other settings, where some concerns around staff turnover, resourcing and institutional base were expressed.

*It would be good to keep the group connected, also find a localized way to expand the conversation, to making it indigenous beyond the trained group, who will be dependent on their jobs. Group conversations are very limited to workshops and meetings—there is no thinking within the group on how to take it forward.* (Pakistan KI)

All teams laid out plans for follow-up actions. Some represented a completion of CAIs planned during the programme (for example, for Myanmar, Niger, Cameroon and Senegal). Others took ambitions further and were more concrete (e.g. for Pakistan and Burkina Faso). Based on the findings in this evaluation, it seems likely that momentum will be sustained in the countries with more concrete plans, which is also indicative of a more specific understanding of blockages and areas with potential for movement.

In relation to overall funding, development partners were supportive, although L4UHC has been heavily dependent financially on two partners and would benefit from diversifying its funding sources and potentially establishing itself as an independent entity, as well as increasing awareness in partners and potential partners of its work.

## Discussion

This evaluation adds important substantive and methodological insights to a field—of global health leadership development—which has relatively few published evaluations. Previous studies have highlighted that there are many obstacles to global healthcare leadership development, such as silo-style disciplinary training, lack of organizational support and cultural differences. Strategies such as critical self-reflection, participatory action learning and developmental evaluation are highlighted as particularly important [20].

In particular, there is limited research on the outcomes of leadership development programmes, particularly at the system level. Individual outcomes, particularly the acquisition of new knowledge and skills, and changes in attitudes and perceptions, are widely captured by leadership programme evaluations, especially those that have occurred in the course of the programme [25]. We have managed to capture a range of indicators, including at the individual, team and outcome level, though we do not focus on the organizational level. Few leadership programmes have developed a theory of change that explicitly links programme activities to short-term and long-term outcomes and impact [17]. In this evaluation, we use a theory-based approach to establish expected pathways to impact, which can be assessed using data from multiple sources. Much of the data were generated through participatory workshops in the course of the programme, and findings were extensively discussed with programme managers and wider stakeholders to validate them and generate consensus around learning and action points arising for the next cycle. We also report programme costs, which is important and rarely done in the academic literature.

Although that has been done previously at the district level [26] and organizational level [27], in this evaluation we use it to assess an ambitious cross-country model in which individuals from a range of sectors are supported to become a network of champions for UHC, with wider linkages to support in the region and internationally. Our evaluation suggests that the model has relevance and has been effective in some domains and contexts. We highlight some of the conditions which appear to have favoured effectiveness in those contexts, such as careful selection of team members (senior but below political level, to be able to influence decisions but still engage with the technical issues); diversity of team membership (including different ages and genders); the critical role of the focal people and coaches who supported the teams; and the structure of the programme, with its encouragement of critical self-reflection, and repeated learning and practice phases. It was also clear from the less successful contexts that getting early institutional buy-in is key, especially in centralized systems.

The combined focus of the programme on adaptive leadership skills and UHC introduced some tensions, particularly at the start of the programme when participants had higher expectations of training in technical aspects of UHC. This highlights the need not only for clearer preparation of participants but also for the programme to connect them with wider UHC resources, as is planned. The programme methodology, which aims to combine personal development with achieving concrete results in-country through collective actions, also can create internal tensions—for example, to maximize changes in impact, the selection of more senior participants may help, but to grow the leaders of tomorrow, it is also important to include some less experienced participants. A diverse team, which includes more and less senior participants, as well as those representing a range of institutional backgrounds, ages and genders, but with a clear mandate to cooperate, may be the most fruitful combination, although each country will present different sensitivities and needs.

Our findings, while specific to this programme, are consistent with the literature on the importance of experiential learning in leadership development [28] and the importance of creating spaces for dialogue, reflection and learning within the health system as a whole [29]. A recent review of global health leadership programmes, for example, also emphasized the challenge of managing hierarchy and diversity of participants, and the value of longer-term support to mentoring, peer networks and horizontal (south–south) learning, which also emerged in our findings [30].

In discussing findings, we need to note a number of important study limitations. The main one is that we generally had to rely on self-reported changes, which is a common challenge in the area of leadership development evaluations. Given that participants were recruited as individuals from a variety of teams, follow-up at the organizational level on behaviour change would not have made sense even if it had been logistically possible. We therefore mitigated risks by triangulating the insights of different parties—not just using data from participants, but also using insights from coaches, CFPs, programme managers, facilitators of modules, and the wider partner group to at least get a broad perspective.

One residual risk, however, is that most respondents (across these groups) were invested to varying degrees in the L4UHC process. This introduces a positive bias. Some mitigation has come from external documents and KIs, at least in two countries, as well as careful interpretation of findings by the evaluators.

Overall response rates for interviews and the surveys were good, but we highlight that it was hard to recruit the full cohort of participants for the final interviews (given its virtual nature, which likely affected willingness to participate). It is possible that those who made themselves available during that final stage were more favourably disposed to the programme.

In addition, there is a general risk of acquiescence bias—that participants would perceive us to be seeking information to validate the programme. We tried to reduce this by phrasing questions in open ways, by emphasizing the independence of the evaluation team, and by showing as much interest in why things do not work as in why they do (and how we can make them better).

It was always foreseen that we would have limited time to follow up on higher-level outcomes and impact; however, this situation was exacerbated by COVID-19, which led to the postponement of Module 3, such that the evaluation had to be finalized with very limited time to study the follow-up to that module. The impact of COVID-19 also meant conducting the country case studies remotely, which reduced their depth and the range of external KIs reached.

The overall conclusions also have to be assessed in relation to a programme that has had to adapt to COVID-19. For a programme focused on adaptive management, there is some logic in expecting it to be capable of flexing to new circumstances. However, the lack of face-to-face contact with participants impacted on programme results, and this atypical context has to be borne in mind in interpreting the results.

## Conclusions

This paper reports on a theory-based evaluation, using contribution analysis and drawing from mixed methods, of a unique leadership development programme focused on adaptive skills for multisectoral leaders of UHC in Francophone Africa and Anglophone Asia. We conclude that L4UHC is an important component in the global health ecosystem, addressing a relevant need with some strong results, especially in relation to individual competencies, team-building across UHC constituencies in-country, and with some concrete gains at national policy and practice level, as well as some positive unintended consequences. The evaluation also highlights areas for strengthening, including extending the engagement of international partners, strengthening links with country and regional training institutions, and building in activities to strengthen links across teams within regions.

Some of the findings, such as the appreciation of the participatory, practical, experiential and exchange components of the programme, the importance of long-term coaching and mentoring, and the value of experiencing health system challenges and solutions in other settings, are likely to be relevant for other leadership development programmes, even those with differing goals and methods.

## Supplementary Information

**Additional file 1.** Evaluation matrix.

## Data Availability

All datasets are available from the authors on reasonable request.

## Abbreviations

CAI: Collective action initiative
CFP: Country focal person
GIZ: Gesellschaft für Internationale Zusammenarbeit
KI: Key informant
L4UHC: Leadership for Universal Health Coverage
MoH: Ministry of Health
OECD-DAC: Organisation for Economic Co-operation and Development–Development Assistance Committee
P4H: Partners for Health
SDC: Swiss Agency for Development and Cooperation
UHC: Universal health coverage
USAID: United States Agency for International Development

## Footnotes

1 https://adaptiveleadershipfoundation.org/post/adaptive-vs-technical-challenges
