# Supplementary material for: A theory-based evaluation of the Leadership for Universal Health Coverage Programme: insights for multisectoral leadership development in global health
Source: Health Res Policy Syst. 2022 Sep 29;20:103. doi: 10.1186/s12961-022-00907-1 (PMC9522438; doi:10.1186/s12961-022-00907-1)
Supplement: Supplementary file 1 — Additional file 1. Evaluation matrix. [file 12961_2022_907_MOESM1_ESM.docx]

Table 1: Evaluation matrix

| Domain/question | Sources | Timing/  frequency | Analysis methods |
| --- | --- | --- | --- |
| Relevance: to what extent does L4UHC address priority needs at different levels? | | | |
| 1.1 Do participants lack capacity to drive forward UHC prior to the programme? Is L4UCH appropriate to their role? Do they perceive this to be a priority? Do they have other opportunities to gain these competences and commitment? What do they see as their learning needs prior to the programme? | In-depth interviews with participants and key resource persons in participating countries  Programme data (self-reflection and assessment)  In-depth interviews with key resource persons in participating countries  Secondary sources (documents; data; focal countries only) | Once, at start of L4UHC cycle  During each module  At end of Module 3 | Qualitative analysis, using deductive and inductive approach  Analysis of trends in indicators; findings of situation analysis on bottlenecks; donor landscape |
| 1.2 At country level, to what extent is leadership a key bottleneck for UHC progress (and what is the evidence for this, versus other bottlenecks)? What programmes exist which address this gap? |  |  |  |
| Effectiveness: to what extent has L4UHC met its objectives? | | | |
| *2.1 What changes to outputs has L4UHC contributed (and how)?* | | | |
| 2.1.1 Specifically, in relation to individual commitment: having a sense of a personal stake in UHC; feeling accountable for moving UHC forward; sense of urgency in relation to UHC | In-depth interviews with participants  Programme data (self-reflection and assessment) | Pre-interviews (first module), compared to post-interviews (third module)  During modules | Qualitative analysis, using deductive and inductive approach |
| 2.1.2 Specifically, in relation to individual competences: stakeholder mapping, listening and dialogue skills, coalition building, change management (ability to negotiate different priorities, achieving shared vision, engage other key stakeholders, adaptive and innovative thinking, collaborative working) |  |  |  |
| 2.1.3 How have participants’ understanding of UHC and how to analyse and engage with it changed as a result of L4UHC? |  |  |  |
| 2.1.4 How were these output changes achieved? What worked well or not (e.g. participant selection; delivery of activities; wider support for teams)? What lessons are there in relation to improving L4UHC’s model/approach and performance in future? | In-depth Interviews with participants | During third module | Qualitative analysis, using deductive and inductive approach |
|  | Participant survey | At the end of each module (three times) | Quantitative analysis |
|  | Programme documents/data (e.g. facilitators notes on feedback at end of each day) | Continuously available | Analysed thematically for implementation issues raised (challenges, solutions, learning during programme) |
|  | In-depth interviews with key resource persons (P4H, host countries, facilitators, etc.) | During first and third modules | Qualitative analysis, using deductive and inductive approach |
| 2.1.5 What other factors may have contributed to changes observed in outputs? | In-depth interviews with participants  Programme data (self-assessment in Module 3; outcome harvesting)  In-depth interviews with key resource persons |  |  |
| *What changes to intermediate outcome has L4UHC contributed (and how)?* | | | |
| 2.2.1 To what extent has L4UHC supported stronger coalitions for UHC at country and regional levels? | In-depth interviews with participants  Programme data (self-assessment in Module 3; outcome harvesting)  In-depth interviews with key resource persons (P4H, participating countries) | Pre-interviews (first module), compared to post-interviews (third module)  During third module | Qualitative analysis, using deductive and inductive approach |
| 2.2.3 How were these outcome changes achieved? What supported or hindered the changes (e.g. institutional factors; contextual factors such as resources or power relationships; individual factors such as motivation or relationships)? What lessons are there in relation to improving L4UHC’s performance in future? |  |  |  |
| 2.2.4 What other factors may have contributed to changes observed in outcomes? |  |  |  |
| *2.3 What changes to higher-level outcome has L4UHC contributed (and how)?* | | | |
| 2.3.1 What changes to UHC policies and practice has the L4UHC contributed in participant countries? | Analysis of CAIs | After third module | Thematic analysis of achievements and their scale versus plans |
|  | In-depth interviews with participants and key resource persons | During third module | Qualitative analysis, using deductive and inductive approach |
|  | Secondary sources (documents—focal countries only) | During site visit (focal countries) | Analysis of changing policy landscape and its determinants |
| 2.3.2 How were they achieved (individual, institutional, contextual factors)? What lessons can we learn from this? | As above for 2.3.1 | | |
| 2.3.4 What other factors may have contributed to these changes? |  |  |  |
| Efficiency: do the outputs and outcomes of L4UHC justify its inputs? | | | |
| 3.1 What resources were planned for and actually absorbed in delivering L4UHC? | Programme budget and expenditure data | Towards end of programme cycle (2020) | Quantitative analysis by broad categories and in relation to other similar programmes, as evidence permits |
|  | Interviews with core management team | Towards end of programme cycle (2020) | Thematic analysis |
| 3.2 Could the programme have been delivered more efficiently with less resource intensity? Conversely, could more have been delivered with the same inputs? How? |  |  |  |
| Impact: what positive and negative changes have been—or are likely to be—achieved by the programme? | | | |
| 4.1 How significant are the changes in policy and practice to which L4UHC has contributed? What is their likely impact on UHC in the longer term? | Analysis of CAIs  Programme data (self-assessment in Module 3; outcome harvesting)  In-depth interviews with participants and key resource persons (including P4H and in participating countries) | After third module  During third module | Thematic analysis, assessed in relation to challenges identified  Qualitative analysis, using deductive and inductive approach |
| 4.2 Are there any unintended effects of the L4UHC programme (positive or negative)? |  |  |  |
| Sustainability: are the benefits of L4UHC likely to endure after the end of the programme? | | | |
| 5.1 How well embedded are the changes at individual and institutional levels to which L4UHC has contributed? | In-depth interviews with participants and key resource persons (including P4H and in participating countries) | During third module | Qualitative analysis, using deductive and inductive approach |
| 5.2 How could their sustainability be better ensured or supported in future? |  |  |  |
